# Inhaled nitric oxide might be a contributing tool for successful resuscitation of cardiac arrest related to pulmonary hypertension

**DOI:** 10.1186/s13049-019-0602-x

**Published:** 2019-02-22

**Authors:** Per P. Bredmose, Christian Buskop, Anne Birthe Lømo

**Affiliations:** 1 Air Ambulance Department, Oslo University Hospital, Postboks 4950 Nydalen, 0424 Oslo, Norway; 2 Department of Obstetrics and Gynaecology, Elverum Hospital, Elverum, Norway

**Keywords:** Inhaled nitric oxide (iNO), Pulmonary hypertension, Amniotic fluid embolus, Cardiac arrest, Resuscitation

## Abstract

We describe a case where inhaled nitric oxide (iNO) was successfully initiated during cardiopulmonary resuscitation (CPR) in a younger patient with cardiac arrest related to pulmonary hypertension after disseminated intravascular coagulation (DIC) postpartum bleeding and hysterectomy. This case illustrates that iNO might be a potential lifesaving tool for resuscitation of patients with cardiac arrest related to pulmonary hypertension, for whom most other resuscitation strategies often are futile.

**Electronic supplementary material:**

The online version of this article (10.1186/s13049-019-0602-x) contains supplementary material, which is available to authorized users.

## Introduction

Cardiac arrest related pulmonary hypertension is a serious condition well known to (and feared by) most resuscitationists. We describe a case where inhaled nitric oxide (iNO) was successfully initiated during cardiopulmonary resuscitation (CPR) in a younger patient with cardiac arrest related to pulmonary hypertension after disseminated intravascular coagulation (DIC) postpartum bleeding and hysterectomy. We believe that this case is of importance in showing the potential to possibly reverse an extreme cardiac arrest situation in a retrieval setting through institution of iNO treatment.

## Background

Cardiac arrest in relation to pulmonary hypertension is a dangerous clinical condition. Amniotic fluid embolism syndrome (AFE) is believed to be initiated when fetal cells enter the maternal circulation resulting in systemic inflammatory response syndrome [1]. This causes pulmonary vasoconstriction, pulmonary hypertension and subsequent failure of the right side of the heart [2]. Also, a coagulopathy may pre-exist or develop secondarily [3]. Resuscitation of patients with pulmonary hypertension is known to be difficult [4]. The cardiac arrest associated hypoxia and hypercarbic acidosis only worsens the situation. It is of critical importance to break this cycle to achieve return of spontaneous circulation (ROSC).

## Case

A 29 year. Old woman presented to a general hospital with intrauterine fetal demise, with sonographic gestational age estimated to 35–36 weeks on arrival. The patient had four older children. On arrival the patient was conscious with a heartrate (HR) of 104 beats/min and blood pressure of 105/83 mmHg. The patient had early signs of Disseminated Intravascular Coagulation [5] with a Hb of 5.4 g/dL, fibrinogen of 1.6 g/L and platelets of 79 × 10^9^/l. After pharmacologic induction of labor, a 2260 g male child was still born. There was spontaneous delivery of the placenta, which showed signs of abruption and bleeding. Following this a large amount of clotted blood was delivered. Subsequently hypotonia of the uterus was recognised. Manual and pharmacological uterotonic maneuvers were instituted with intravenous and intramuscular oxytocin and rectal misoprostol. The patient was taken to the operating theatre for further haemorrhage control. Parallel to this a total of three units of packed red blood cells and 2000 ml of Ringers acetate were given. After induction of general anaesthesia with propofol, fentanyl and suxamethonium there was a fall in invasively measured BP and at a systolic blood pressure of 30 mmHG CPR was started. The cardiac arrest lasted only shortly and was treated with conventional measures according to guidelines for advanced resuscitation [6]. Initial control of the bleeding was attempted with methylergomethrine and carboprost as well as an intra-uterine-balloon. Due to lack of haemostasis a total hysterectomy was performed. During the surgery the patient suffered one more episode of pulseless electrical activity (PEA) treated with adrenaline and CPR as well as an episode of ventricular fibrillation treated with two DC shocks and CPR. An ultrasound performed during surgery showed a partly empty left ventricle and a grossly dilated right ventricle. The patient was taken to the Intensive Care Unit (ICU) after having received a total of eleven units of erythrocytes, five units of plasma and two units of thrombocytes as well as tranexamic acid and fibrinogen. The patient was intubated, ventilated and sedated on arrival in the ICU. High doses of intravenous noradrenaline (0.7 micrograms/kg/min) in addition to adrenaline (0.1 micrograms/kg/min) were continued from the operating room to the ICU. In the ICU the patient was connected to a ventilator on 80% oxygen. A transthoracic echocardiogram was performed by a local cardiologist, which showed moderate pulmonary hypertension without detailed estimates of pressure values. At this stage differential diagnoses were amniotic fluid embolism (AFE), acute respiratory distress syndrome, DIC or sepsis.

In order to facilitate transport to a university hospital, a specialised transport team from the Air Ambulance department at Oslo University Hospital was requested. Due to severe weather conditions one team (flight physician and transport nurse) was sent by road in an ICU ambulance and parallel to that, a team consisting of flight physician and Helicopter Emergency Medical Service (HEMS) crew member and pilot was sent by helicopter (EuroCopter 145). The latter was carrying equipment for initiating treatment with inhaled nitric oxide (iNO). Upon arrival of the HEMS retrieval team, the patient was ventilated with high pressures on the ventilator (Pmax 27 cm H_2_O) and positive end expiratory pressure (PEEP) of 17 cm H_2_O.

On the basis of the acute right ventricular failure and pulmonary hypertension, nitric oxide was planned introduced before initiation of the transport. The physician of the transport team performed a transthoracic ultrasound examination shortly after arrival [7]. The ultrasound examination showed a grossly enlarged right ventricle and right atrium and a globally poor contraction status of the heart. An additional movie file shows this in more detail [see Additional file 1]. Shortly after the ultrasound examination the oxygen saturation started dropping. This was regarded as early sign of a reduction of pulmonary blood flow and a new ultrasound examination was performed, showing globally grossly reduced contractions as seen in a peri-arrest situation. An additional movie file shows this in more detail [see Additional file 2]. FiO2 was increased to 1.0. Shortly after the patient developed a cardiac arrest (PEA) with an invasive BP that declined. After a minute, the patient went via PEA into asystole. High quality CPR with cardiac compressions and manual ventilation on the endotracheal tube with 100% oxygen was immediately started. Meanwhile a manual system (InoVent GE Healthcare, Milwaukee, WI, USA) for delivery of iNO was rapidly assembled and iNO ventilation was initiated with a bag on the endotracheal tube less than five minutes into the cardiac arrest with 20 ppm iNO. After one minute with iNO ventilation, ROSC was confirmed on invasive BP monitoring and a rise in End-Tidal Carbon Dioxide (ETCO2). Three minutes after ROSC a new ultrasound showed normalisation of right side of the heart and global improvement of contractility. An additional movie file shows this in more detail [see Additional file 3]. The patient was then transported with continuous iNO treatment en route with a portable iNO delivery apparatus (InoVent GE Healthcare, Milwaukee, WI, USA) and a LTV1200 transport ventilator (Carefusion, San Diego, CA, USA) to the university hospital. The 40 min transport by helicopter was uneventful despite the patient being in an unstable condition.


Additional file 1: Pre-cardiac arrest ultrasound. Parasternal long axis ultrasound showing grossly enlarged right atrium and right ventricle. Also, reduced low filling volumes in both left atrium and left ventricle, as well as compensating tachycardia are seen. (MP4 6635 kb)



Additional file 2: Peri-cardiac arrest ultrasound. Parasternal long axis ultrasound showing a pre-arrest situation with critically reduced low filling volumes, globally reduced contractility and accompanying bradycardia. Right chambers of the heart are grossly enlarged. (MP4 9075 kb)



Additional file 3: Post return of spontaneous circulation ultrasound after initiation of inhaled nitric oxide. Parasternal long axis ultrasound with improved filling of both left atrium and ventricle and good contractility globally. The previously enlarged right side of the heart has normalised in size. (MP4 6341 kb)


After ICU treatment the patient made a complete physical and neurological recovery with no cognitive sequelae.

## Discussion

We were unable to find publications describing initiation of iNO at a remote hospital as a selective pulmonary vasodilator during resuscitation of a cardiac arrest in a patient with DIC and AFE. Our case describes its successful initiation and use. We have found one other case of iNO initiation during cardiac arrest within the hospital [8].

Resuscitation of the deteriorating patient with pulmonary hypertension which subsequently goes into cardiac arrest is a challenge [9].

Established treatments for pulmonary hypertension are the following; improving oxygenation, alkalisation, hyperventilation (without hyperinflation) and reassuring that hypovolemia is not present [10]. These are all treatments that can be instituted at almost any ICU. Also, sildenafil, epoprostenol and inhaled nitroglycerin have been used [11]. However, one of the most potent vasodilators acting selectively on the pulmonary circulation is iNO [12].

The cardiac arrest treatment algorithm is well known and well implemented in most hospitals. If pulmonary hypertension is a potential background for cardiac arrest, treatment according to the conventional algorithm is less likely to succeed. The reversible causes, commonly known as the “4 H’s and 4 T’s” should be addressed [6]. However, in the setting we describe, achievement of ROSC seems to be dependent on re-establishment of pulmonary blood flow (reducing resistance) and reducing the strain on right ventricular function.

The Air Ambulance department of Oslo University Hospital is based on sending consultants in retrieval medicine and prehospital care (senior consultant anesthesiologists which is a combined specialty in Norway with intensive care and critical emergency medicine) to the patient. In this case, this was an urgent secondary inter-hospital transport. Due to the background information about the patient and medical history, portable medical technical equipment for delivering iNO was brought to the patient. The understanding of such extreme physiological conditions is an important condition for undertaking such transports. Such transports aren’t “just transport”, but “bringing the university hospital closer to the patient”. The Air Ambulance department at Oslo University Hospital has an already well-established practice for both initiation of iNO treatment as well as providing continuous iNO treatment during the retrieval phase [13].

Institution of iNO treatment has been described previously for treatment of pulmonary embolus, pulmonary hypertension and AFE [8, 14]. In this case the ability to perform and evaluate a transthoracic ultrasound rapidly both confirmed the diagnosis and the response to iNO treatment.

In many hospitals iNO is available for treatment in ICU. In many neonatal units there might be access to iNO treatment. In urgent critical situations “thinking outside the box” and utilising such medical equipment could be lifesaving.

We believe that the initiation of iNO treatment was the intervention that made achievement of ROSC possible. The pre and post ultrasound shows the physiological improvement especially on the right side of the heart. Other cases of AFE have been treated with extracorporeal membrane oxygenation [15, 16]. This is to our experience a more time and resource consuming procedure in the retrieval situation.

The majority of hospitals do not have this treatment option, although some neonatal units might have it available. This is a tool that should be considered for resuscitation of AFE in either arrest or peri-arrest. In this case, the availability of a highly skilled and well-developed physician-based retrieval system seems to have been a contributor to the successful outcome in this case. For the retrieval physician the case illustrates the importance of relevant in-hospital duties and the ongoing experience with advanced intensive care medicine.

Therefore we recommend that in cardiac arrest related to pulmonary hypertension focus is kept on good quality CPR, hyperoxygenation and rapid reduction in pulmonary resistance.

## Conclusion

This case illustrates that iNO is a potential lifesaving tool for resuscitation of patients with cardiac arrest related to pulmonary hypertension, for whom most other resuscitation strategies often are futile.

In this case the combination of DIC, bleeding and hysterectomy might have led to AFE with pulmonary hypertension [1]. High quality resuscitation and early institution of iNO might have been the cause of a successful outcome for this patient. Having such possibility in an advanced retrieval system staffed with senior physicians with long experience in ICU seems useful in some patient categories. We therefore believe that iNO is a treatment that could be in the toolbox of advanced retrieval systems.
